# The associations between unhealthy behaviours, mental stress, and low socio-economic status in an international comparison of representative samples from Thailand and England

**DOI:** 10.1186/1744-8603-10-10

**Published:** 2014-02-20

**Authors:** Antonio Ivan Lazzarino, Vasoontara Yiengprugsawan, Sam-ang Seubsman, Andrew Steptoe, Adrian C Sleigh

**Affiliations:** 1 Department of Epidemiology and Public Health, University College London, 1-19 Torrington Place, London WC1E 6BT, UK; 2 National Centre for Epidemiology and Population Health, The Australian National University, Acton, Canberra, ACT 2601, Australia; 3 School of Human Ecology, Sukhothai Thammathirat Open University, Pakkret, Nonthaburi 11120, Thailand

**Keywords:** Psychological stress, Psychosocial deprivation, Health behavior, Social class

## Abstract

**Background:**

Socioeconomic status is a recognised determinant of health status, and the association may be mediated by unhealthy behaviours and psychosocial adversities, which, in developed countries, both aggregate in low socioeconomic sectors of the population. We explored the hypothesis that unhealthy behavioural choices and psychological distress do not both aggregate in low socioeconomic status groups in developing countries.

**Methods:**

Our study is based on a cross-sectional comparison between national population samples of adults in England and Thailand. Psychological distress was assessed using the General Health Questionnaire (GHQ-12) or three anxiety-oriented items from the Kessler scale (K6). Socioeconomic status was assessed on the basis of occupational status. We computed a health-behaviour score using information about smoking, alcohol consumption, fruit and vegetable consumption, and physical activity.

**Results:**

The final sample comprised 40,679 participants. In both countries and in both genders separately, there was a positive association between poor health-behaviour and high psychological distress, and between high psychological distress and low socioeconomic status. In contrast, the association between low socioeconomic status and poor health-behaviour was positive in both English men and women, flat in Thai men, and was negative in Thai women (likelihood ratio test P <0.001).

**Conclusion:**

The associations between socioeconomic status, behavioural choices, and psychological distress are different at the international level. Psychological distress may be consistently associated with low socioeconomic status, whereas poor health-behaviour is not. Future analyses will test whether psychological distress is a more consistent determinant of socioeconomic differences in health across countries.

## Introduction

Socioeconomic status is a recognised determinant of health status. In developed countries, the lower a person’s socioeconomic status the worse his or her health. Even in the most affluent countries, people from lower socioeconomic status groups have considerably shorter life expectancies and greater morbidity than people from higher socioeconomic status groups [1-3]. Lower socioeconomic status is associated with greater risk of coronary heart disease, hypertension, diabetes, and other non-communicable diseases in developed countries such as England [4]. Many factors contribute, but among the most prominent candidates are poor health-related behaviours and psychosocial adversity [1-3].

Behaviours such as smoking, food choice, physical inactivity, alcohol consumption are well recognised determinants of health [5]. Psychosocial adversities such as chronic stress, low social support, depression, marital conflicts, work-related stress, financial strain and others have been less well studied, and the evidence is less consistent. However, meta-analyses of prospective observational studies indicate that stress from work and other sources predicts future coronary heart disease [6] and stroke [7], while depression and psychological distress are associated with future diabetes [8] and some forms of cancer [9].

There is difficulty in disentangling the relative impact of health-behaviour and psychosocial adversity on socioeconomic status differences in the health status of people living in developed countries. The reason is that in the USA, Europe and other high-income countries, lower socioeconomic status is associated both with poor health-behaviour and with the accumulation of psychosocial adversity [10,11]. The relative importance of psychosocial and health-behaviour explanations of socioeconomic status differences in health has attracted considerable debate [12,13].

However, the profile may be different in middle and lower income countries, leading to the possibility of assessing the differential contributions of psychosocial and health-behaviour pathways. Illnesses that are linked with poor hygienic conditions, malnutrition, and lack of health care, such as acute diarrhoeal diseases, acute lower respiratory infections, and tuberculosis, are in the top ten causes of death. Although non-communicable health problems such as coronary heart disease, diabetes and obesity are becoming increasingly important in middle and lower income countries, they continue to have less impact on the health status of the population [14,15].

Thailand is a country in epidemiological transition and disease patterns are now beginning to resemble those in developed countries, reflecting the socioeconomic and environmental transformations and associated changes in risks to health. There is evidence for increasing rates of non-communicable diseases. For example, heart disease admissions increased from 56.5 to 397.0 per 100,000 between 1985 and 2003, a seven-fold increase. Diabetes has increased from 33.3 to 380.7 per 100,000 over the same period, an eleven-fold increase [16,17].

It is likely that the profile of cardiovascular risk is changing in the Thai population [18]. Cardiovascular risk factors are greater in the urban than rural population, though socioeconomic status risk profiles are somewhat mixed. For example, smoking is inversely related to income in men and positively related to income in women [19], but obesity is positively related to socioeconomic status in men and negatively in women [20]. Self-reported morbidity shows a similar relationship with socioeconomic status in Thailand as in the UK, with greater rates among lower socioeconomic status individuals [21], but emerging data indicate that low socioeconomic status is a determinant of greater cardiovascular mortality [22]. Very little is currently known about the socioeconomic distribution of psychosocial risk factors, and how they relate to socioeconomic status in Thailand.

Gender differences in patterns linking mental health with health behaviours have been documented in Europe too, where a World Health Organization 2009/10 survey described how in general males externalize more the psychological adversities they encounter with expressive forms of health behaviours, such as smoking and drinking for example, while females tend to internalize their emotions, often manifesting psychosomatic symptoms or mental health problems [23].

### Aim of the study

A comparison between England and Thailand may provide an opportunity to distinguish between psychosocial and health-behaviour explanations for socioeconomic status differences in health. We therefore set out to compare the links between socioeconomic status, behavioural choices, and psychosocial risk factors in England and Thailand, to explore the hypothesis that unhealthy behavioural choices and psychological distress do not aggregate in low socioeconomic status groups of both countries.

## Methods

This study is based on a cross-sectional comparison between representative population samples of adults in England and Thailand: the Health Survey for England and the Thai Cohort Study.

### The databases

The Health Survey for England (HSE) is a nationally representative, general population-based study, recruiting individuals living in private households in England using stratified random sampling. HSE comprises a series of annual surveys beginning in 1991 and it is designed to provide regular information on various aspects of the nation’s health. All surveys have covered the adult population aged 16 and over living in private households in England. The information is collected during household visits by trained investigators using Computer-Assisted Personal Interviewing (CAPI). The HSE has a set of core elements that are measured every year and special topics that are measured in selected years. Core topics include: general health; smoking; drinking; fruit and vegetable consumption; height; weight; blood pressure; blood and saliva parameters. Special topics include: cardio-cerebrovascular disease; physical activity; accidents; lung function; special blood parameters; eating habits; oral health; asthma. In addition, psychosocial factors such as stress, social relationships and depression are assessed [24]. We have used HSE data from years 2003 and 2004.

The Thai Health Risk Transition Study (THRT) includes a large national cohort study of distance-learning Open University students of modest means and representing trends that will become more general among Thais in the future. Because the members of this Thai Cohort Study (TCS) are well educated they are capable of responding to sophisticated questionnaires To generate TCS, in 2005 the distance-learning students from Sukhothai Thammathirat Open University (STOU) residing all over Thailand were mailed a 20-page questionnaire covering socio-demographic details, local environment, income and work, health, injuries and health service use, social networks and well-being, diet, physical activity, and tobacco and alcohol consumption. The TCS includes a second follow-up questionnaire in 2009 with a third follow-up scheduled for 2013, but the present study is restricted to the 2005 baseline survey [16]. The TCS is representative of the geo-demographic, ethnic composition, religion, income and household assets of the adult Thai population. Based on the results of the 2000 Population and Housing Survey, the median age was 29.2 years for the Thai population and 29.0 years among cohort members, and 51% of the Thai population were women compared with 54% of cohort members [25,26]. We can further note that the TCS represent well institutionalised Thais including those residing in monasteries, police and military dormitories, and prisons.

### Measures and data harmonization

Full details of the measures in the two countries and their harmonization for analytic purposes are presented in the Additional file 1. Briefly, socioeconomic status was assessed on the basis of occupational status, and participants were divided into three categories (high, medium and low) in each country. Psychological distress was assessed using the General Health Questionnaire (GHQ-12) in England [27,28], and three anxiety-oriented items from the Kessler scale (K6) in Thailand [29,30]. Both sets of questions referred to psychological experience over the past four weeks. We created a harmonised variable with three categories: high, medium and low distress. As for health-behaviour, the following variables considered: smoking; alcohol consumption; fruit and vegetable consumption; physical activity. We computed a health-behaviour score by allocating one point for each of the following conditions: current smoking; regular alcohol consumption; less than three servings of vegetables or fruit per day; less than two sessions of moderate or vigorous physical activity per week. The health-behaviour score was therefore structured with five categories ranging from zero (good health-behaviour) to four (poor health-behaviour). This kind of score has been used and validated as a predictor of total cause, CVD, and cancer mortality in previous studies [31,32]. We then cut the score into three categories: score zero = good health-behaviour; score one = medium health-behaviour; scores from two to five = poor health-behaviour. Therefore all our final main variables indicating socioeconomic status, psychological distress, and health-behaviour took the format of ordered categorical variables structured with three categories each.

### Data analysis

We firstly describe the sample after stratification by country and gender. Afterwards, for each of the four strata (Thai women, Thai men, English women, and English men) we analysed the correlations between socioeconomic status, psychological distress and health-behaviour using Spearman’s Rho. The associations were further analysed using logistic regression. We fitted three separate models: in the first model we considered psychological distress as a function of health-behaviour; in the second model we considered socioeconomic status as a function of psychological distress; in the third model we considered health-behaviour as a function of socioeconomic status. For each logistic regression model the outcome was transformed into binary variable and we used the following cut points: good/medium health-behaviour versus poor health-behaviour; low psychological distress versus medium/high psychological distress; high/medium socioeconomic status versus low socioeconomic status. Each model was adjusted for age and stratified by country and gender. We then calculated age-standardised outcome probabilities for each stratum using linear prediction on the log-odd scale, i.e. predicted log-odds were transformed into probabilities. Predicted probabilities and 95% confidence intervals from each model were then plotted.

For each logistic regression model we used the Likelihood Ratio Test (LRT) to assess gender-specific and country specific interactions adjusted for age. So, we tested whether the strength of association (gradient of the regression line) between exposure and outcome differed according to gender or country regardless the effect of age. For example, for a comparison between Thai men and women we ran a model with age and gender as covariates restricted to the Thai subsample; estimates were saved; the model was then run again adding in an interaction parameter between the exposure variable and gender; the estimates from this second model were then compared with the estimates of the previous model using the LRT. The LRT is reliable when estimates are made on the same observations (missing values can distort LRT results) and this assumption was always satisfied.

Finally, we carried out sensitivity analyses on missing values. The entire analysis was rerun four times: once after having recoded socioeconomic status missing values to High, once after having recoded socioeconomic status missing values to Low, once after having recoded psychological distress missing values to High, and once after having recoded psychological distress missing values to Low.

### Ethics

Participants in the Health Survey for England gave full informed consent, and ethical approval was obtained from the London Research Ethics Committee. In the Thai study, Ethics approval was obtained from Sukhothai Thammathirat Open University Research and Development Institute (protocol 0522/10) and The Australian National University Human Research Ethics Committee (protocol 2004344). Informed written consent was obtained from all participants.

## Results

The final sample comprised 40,679 participants (24,743 from THRT and 15,936 from HSE). Overall, 43.7% of the English subsample was male, as opposed to the 56.5% of the Thai subsample (Chi^2^-test P value <0.001). English participants were on average 15 years older than Thai participants (T-test P value <0.001). Table 1 shows summary statistics for all variables after stratification by country and gender (Thai women, Thai men, English women, and English men). Thai women had very low prevalence of tobacco and alcohol consumption compared to the other three groups (Chi^2^-test P values <0.001). Both Thai men and women had 20% missing values for socioeconomic status. Both English men and women had 7% missing values for psychological distress.

**Table 1:** Health survey for England (2003–4) and Thai cohort study (2005): characteristics of the study samples

**Factor and category**	**Thailand**		**England**	
	**Women**	**Men**	**Women**	**Men**
N.	10,772	13,971	8,966	6,970
Age (mean ± s.d.)	40.4 ± 4.9	42.0 ± 6.2	56.7 ± 14.7	56.3 ± 13.8
Current smoker (%)	1.2	21.0	20.8	20.3
Current drinker (%)	1.0	12.4	17.3	28.7
Scarce consumption of Fruit and vegetables (%)	26.3	38.5	22.1	25.6
Scarce physical activity (%)	51.1	29.6	73.7	70.8
Socioeconomic status (%)				
1 - High	29.9	31.9	31.3	40.7
2 - Medium	47.3	36.5	41.4	41.9
3 - Low	22.8	31.6	27.3	17.4
Psychological distress (%)				
1 - Low	48.0	45.7	62.0	67.2
2 - Medium	40.0	43.8	23.4	21.2
3 - High	12.0	10.5	14.6	11.6
Health-behaviour (%)				
1 - Good	38.8	35.1	15.2	14.4
2 - Medium	43.3	36.6	47.6	41.3
3 - Poor	17.9	28.3	37.2	44.3

Table 2 shows the results of the correlation analyses using Spearman’s Rho. Poor health-behaviour was positively correlated with psychological distress in all four strata, with the four coefficients being similar (they range from 0.07 to 0.09) and all P values being <0.001. Psychological distress was also positively correlated with low socioeconomic status in all strata, but the association did not reach statistical significance in Thai women. The correlation between socioeconomic status and health-behaviour differed between countries: in England, low socioeconomic status was correlated with poor health-behaviour, showing similar coefficients between genders (for men = 0.15, for women = 0.14) and P values <0.001; in Thai men, low socioeconomic status was also positively correlated with poor health-behaviour, but only weakly (Rho = 0.02, P = 0.113); in Thai women, the correlation went in the opposite direction compared with the English sample: lower socioeconomic status was negatively correlated with poor health-behaviour (Rho = −0.04, P = 0.002).

**Table 2:** Spearman’s rho correlation coefficients and P values (in brackets) between psychological distress (PD), socioeconomic status (SES) and health-related behaviour (HB)

		**Thailand**			**England**		
		**PD**	**SES**	**HB**	**PD**	**SES**	**HB**
**Female**	PD	1			1		
	SES	0.01	1		0.02	1	
		(0.422)			(0.034)		
	HB	0.09	−0.04	1	0.08	0.14	1
		(<0.001)	(0.002)		(<0.001)	(<0.001)	
**Male**	PD	1			1		
	SES	0.06	1		0.03	1	
		(<0.001)			(0.010)		
	HB	0.09	0.02	1	0.07	0.15	1
		(<0.001)	(0.113)		(<0.001)	(<0.001)	

All variables are structured in three ordered categories.

**Figure 1:**
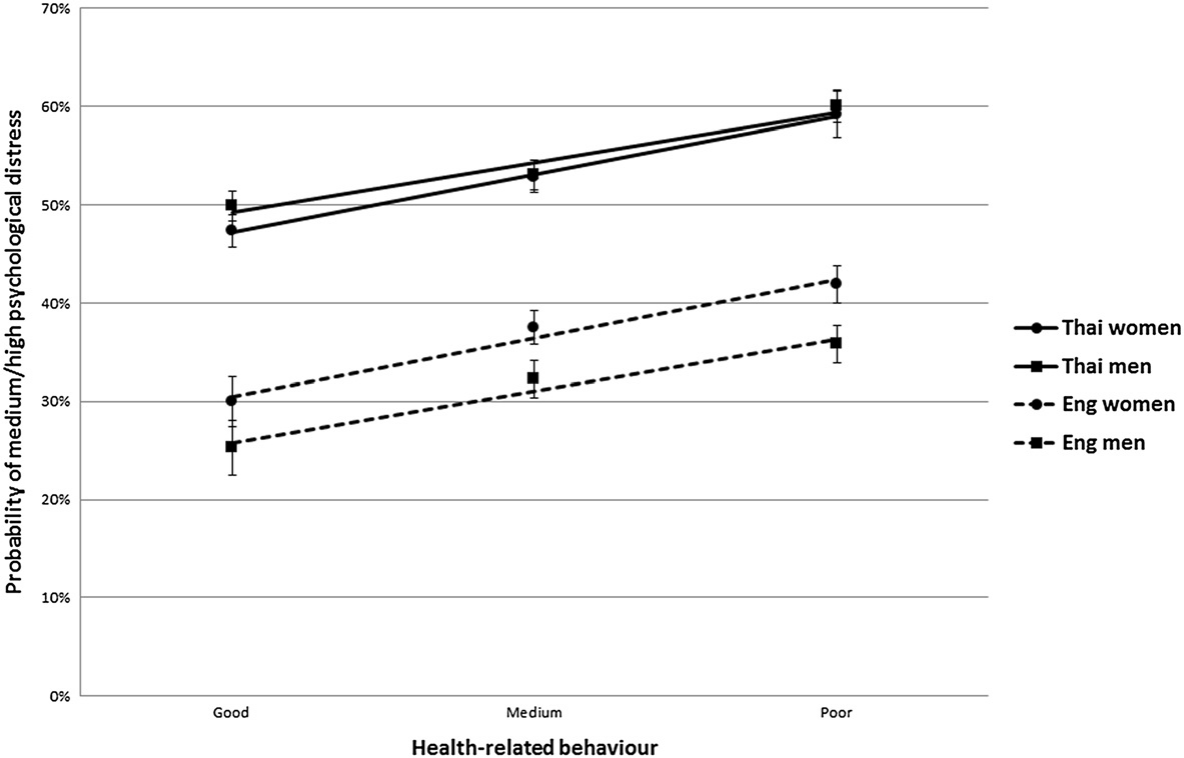
**Age-standardised probability of medium/high psychological distress as a function of health-behaviour, stratified by country and gender, with 95% confidence intervals.** LRT P values for interaction (different gradient between lines): Thai women vs English women: P = 0.174; Thai men vs English men: P = 0.174; Thai women vs Thai men: P = 0.698; English men vs English women: P = 0.781.

Figure 1 shows the association between psychological distress and health-behaviour, and illustrates the age-standardised probabilities of medium or high psychological distress prevalence as a function of health-behaviour, after stratification by country and gender. The Thai subsample showed higher levels psychological distress, while in England women were more likely to report psychological distress than men. The association between health-behaviour and psychological distress was similar in each stratum, with people with poor health-behaviour having higher chances of medium/high psychological distress. The LRT showed no evidence of effect modification between strata (Thai women vs English women: P = 0.174; Thai men vs English men: P = 0.174; Thai women vs Thai men: P = 0.698; English men vs English women: P = 0.781).

**Figure 2:**
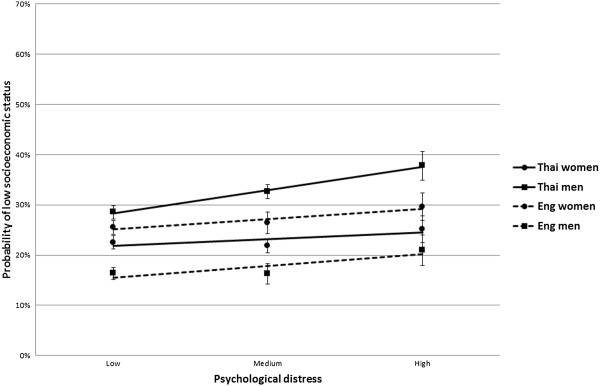
**Age-standardised probability of low socioeconomic status as a function of psychological distress, stratified by country and gender, with 95% confidence intervals.** LRT P values for interaction (different gradient between lines): Thai women vs English women: P = 0.197; Thai men vs English men: P = 0.197; Thai women vs Thai men: P = 0.002; English men vs English women: P = 0.988.

Figure 2 shows the association between socioeconomic status and psychological distress, and the age-standardised probabilities of low socioeconomic status as a function of psychological distress, after stratification by country and gender. In both countries, there was an association between psychological distress and lower socioeconomic status, with greater prevalence of lower socioeconomic status in participants reporting higher psychological distress. The Likelihood Ratio Test showed evidence of effect modification between Thai women and Thai men only (P = 0.022), with a stronger association between psychological distress and lower socioeconomic status in men than women. The other tests of effect modification were not significant (Thai women vs English women: P = 0.197; Thai men vs English men: P = 0.197; English men vs English women: P = 0.988).

**Figure 3:**
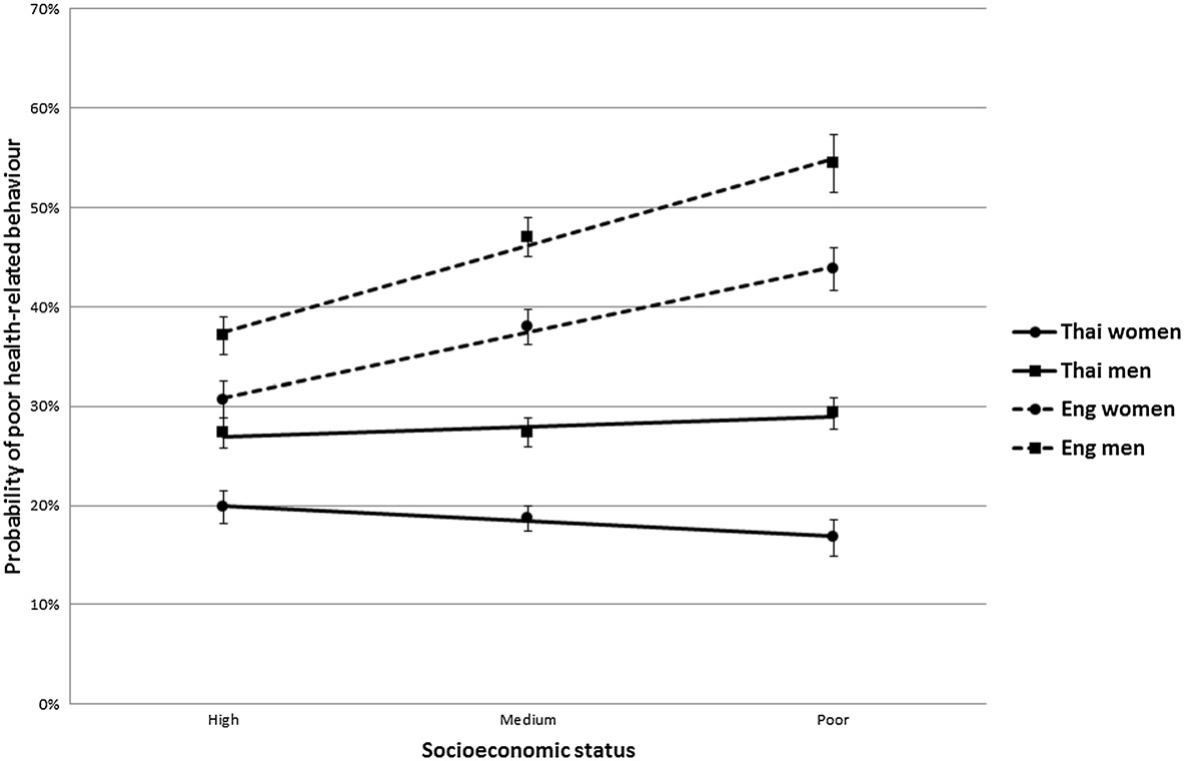
**Age-standardised probability of poor heath behaviour as a function of socioeconomic status, stratified by country and gender, with 95% confidence intervals.** LRT P values for interaction (different gradient between lines): Thai women vs English women: P < 0.001; Thai men vs English men: P < 0.001; Thai women vs Thai men: P = 0.003; English men vs English women: P = 0.126.

The association between poor health-behaviour and socioeconomic status is shown in Figure 3, where age-standardised probabilities of poor health-behaviour are plotted as a function of low socioeconomic status, after stratification by country and gender. There was a lower prevalence overall of poor health-behaviour in the Thai than English sample. In both countries, men reported poorer health-behaviour than women, and the gender difference was only slightly greater among the poor in both settings. In addition, there was a strong country difference in the relationship between socioeconomic status and health-behaviour. In both men and women from England, there was a sharp social gradient, with the prevalence of poor health-behaviour increasing with lower socioeconomic status. By contrast, no relationship between socioeconomic status and poor health related behaviours was evident in Thai men, while in Thai women a moderate reverse gradient was apparent, with lower socioeconomic status women reporting better health-behaviour. With the exception of the within-England gender comparison, the Likelihood Ratio Test showed very strong evidence of effect modification between strata (Thai women vs English women: P < 0.001; Thai men vs English men: P < 0.001; Thai women vs Thai men: P = 0.003).

The sensitivity analyses produced very similar results, with strong associations between socioeconomic status and health-behaviour in English men and women, and Thai participants showing flat (men) or negative (women) associations between socioeconomic status and health-behaviour.

## Discussion

We have shown that the relationships between low socioeconomic status and unhealthy behaviours and high levels of psychological distress not always go hand-in-hand. In England, lower socioeconomic status was associated with both higher psychological distress and poorer health-behaviour, whereas in Thailand lower socioeconomic status was related to greater psychological distress but not to poorer health-behaviour. In both settings, females were substantially better protected against poor health behaviours than their male counterparts but we note that overall health behaviours were generally better in Thailand and were essentially unrelated to social class. This result is compatible with other studies from low-income countries [33,34]. In contrast, respondents in England showed less healthy behaviours associated with advancing poverty, as has been previously shown in other studies in Western countries [12,13]. Indeed, there was evidence in Thai women that higher socioeconomic status was associated with poorer health-behaviour. This was reflected in the TCS data by the previously described positive association between SES and smoking for females, with smoking rates rising from 0.9% to 2.5% as income increases from lowest to highest [19]. The transition from rural to urban living in Thailand is also linked with adverse changes in food choice and physical activity [35].

We have also observed that the occurrence of psychological distress was unrelated to gender among Thais (narrowing gender differences across levels of health-related behaviours); this result is consistent with another study from Japan [36] and may reflect a common cultural pattern in Asian countries. In contrast, among the UK samples, females reported significantly more distress at any given level of health related behaviours, which is consistent with a 2009/10 World Health Organization survey in Europe [23].

The differences in associations between socioeconomic status and behaviour and psychological distress between countries provide opportunities for teasing out the contributions of behavioural and psychosocial factors to social inequalities in health. Cross-country comparisons are valuable for helping identify what relationships appear to be universal and which depend on local contextualizing factors. Portugal has concluded a rapid transition to political democracy occurring in the seventies, which resulted in a rapid improvement in living standards, essentially marked by an increased access to consumer’s goods, not always accompanied by parallel social and cultural changes, particularly among the lowest SEP group [37]. In a recent study on socioeconomic inequalities in Portugal, better health behaviour and lower levels of depression were registered in higher SES groups [37].

### Strengths

The strength of our study is its large national scale with around 40,000 adults representing young and middle-aged Thai and English adults residing throughout the countries. The data selection and analysis strategy had been agreed between the two research-teams before any kind of data analysis or dataset merging was carried out, and this excludes limitations such as ‘result fishing’ or ‘fitting the data to the hypotheses’. The questionnaires used in the two surveys were very similar and the data harmonisation prior to the combined analysis was not difficult to perform.

### Limitations

This study is based on the analysis of data collected separately in two different countries. Therefore there could be issues of information bias. However our variables of interest are not country specific and have been consistently identified in population surveys as relevant to health in different parts of the world. In fact database harmonisation was not difficult to perform since all variables had comparable categories.

The study is cross-sectional and therefore we cannot eliminate issues of reverse causality, except for the many attributes which are unchanging (e.g., skin or eye colour, native language) or fixed from late childhood (e.g., attained height). However, the aim of the study was not to establish causal relationships towards any clinical outcome, but rather to establish whether different patterns of association between low socioeconomic status, health-behaviours and psychological distress are present in the two countries.

Selection bias can be an issue if differential recruitment to the study occurred in the countries. Missing values can also introduce selection bias; however the sensitivity analysis showed that our results are robust.

### Future research

More explicit accounting for culture would help reveal the causal web underlying our principal question — the linkage (or non-linkage) between socioeconomic status and adverse health behaviours and psychosocial outcomes. It is probable that an ethnographic approach would help deepen the conceptual model which is beginning to form and able to detect and elaborate the action of culture going beyond the limits of our study.

## Conclusion

In Thailand, during the epidemiological transition, the pattern linking socioeconomic status, behavioural choices, and psychological risk factors is different from England, with unhealthy behaviours and high levels of psychosocial adversities not both aggregating in lower socioeconomic status sectors of the population.

Psychological distress is more consistently associated with low socioeconomic status than poor health-behaviour is across countries such as England and Thailand. Future analyses will test whether psychological distress is a more consistent determinant of socioeconomic differences in health across countries.

## Competing interests

The authors declare that they have no competing interests of any kind.

## Authors’ contributions

AL and VY had full access to the data, and take responsibility for the integrity of the data and accuracy of the data analyses. AL conducted analyses. All authors contributed to the concept and design of study, drafting, and critical revision of the manuscript. All authors read and approved the final manuscript.

## Supplementary Material

Additional file 1 Appendix for the article titled “Relationships among psychosocial risk factors, health-related behaviours, and socioeconomic status”. Click here for file
