# Supplementary material for: The associations between unhealthy behaviours, mental stress, and low socio-economic status in an international comparison of representative samples from Thailand and England
Source: Global Health. 2014 Feb 20;10:10. doi: 10.1186/1744-8603-10-10 (PMC3933467; doi:10.1186/1744-8603-10-10)
Supplement: Additional file 1 — Appendix for the article titled “Relationships among psychosocial risk factors, health-related behaviours, and socioeconomic status”. [file 1744-8603-10-10-S1.docx]

**Appendix for the article titled “Relationships among psychosocial risk factors, health-related behaviours, and socioeconomic status.”**

**Data harmonisation**

The two datasets underwent a cleaning procedure: inconsistent, duplicate, outlier, and missing values were checked, as well as digit preferences. Normality of each continuous variable was checked.

Socioeconomic status

We used the profession of the individual as an indicator of socioeconomic status (SES). As for England, the Registrar General’s Social class is a social classification system that attempts to classify groups on the basis of employment, based on characteristics such as career prospects, autonomy, and mode of payment and period of notice. The Health Survey for England uses a six category system for occupation in which informants are classified as managerial and professional, intermediate, small employers and own account workers, lower supervisory and technical, and semi-routine and routine occupations. We found a parallel measure of socioeconomic status based on profession used in the Thai Cohort Study, which is also structured in six categories: Professionals, Senior managers, Middle Managers, Office Assistants, Skilled Workers, Manual Workers. We created a harmonised variable structured with three categories. For England, we collapsed the six categories as follows: Professional / Managerial Position = High SES; Skilled Manual / Non-Manual Workers = Medium SES; Semi-Routine / Unskilled Workers = Low SES. For Thailand, we collapsed the six categories as follows: Professionals / Senior / Middle Managers = High SES; Office Assistants = Medium SES; Skilled Workers / Manual Workers = Low SES.

Psychological stress

The Health Survey for England uses the General Health Questionnaire (GHQ-12) to measure psychological stress. The GHQ-12 is generally considered to be a uni-dimensional scale, and consists of twelve items relating to anxiety, depression, social dysfunction, and loss of confidence. Interpretation of each of the 12 answers is based on a four point response scale that we collapsed to a binary score (symptom present: “not at all” or “same as usual” = 0; “more than usual” or “much more than usual” = 1). The questionnaire therefore gives a total score for psychological stress that varies from zero to twelve. In the Thai Health-Risk Transition study, psychological stress was assessed using the three anxiety-oriented items of the Kessler 6 psychological stress questions previously used in the US National Health behavior Survey. The questions we used were: “In the past 4 weeks, about how often did you feel: 1) nervous; 2) restless or fidgety; 3) everything was an effort”. Answers to each of these questions were on a 5-point scale ranging from 1 (“all of the time”) to 5 (“none of the time”). The questionnaire therefore gives a total score for psychological stress that goes from three to fifteen. We created a new harmonised variable structured with three categories. For England, we collapsed the 13 categories (from 0 to 12) of the GHQ12 score into three categories: GHQ score zero = low level of stress; GHQ scores 1 to 3 = medium level; scores 4 or more = high level. For Thailand, we collapsed the 13 categories (from 3 to 15) of the Kessler score into three categories: scores 10 to 15 = low level; scores 8 to 9 = medium level; scores 3 to 7 = high level.

Smoking

The Health Survey for England and the Thai Health-Risk Transition study derived variables having the same categorisation structure: current cigarette smoker; ex-regular cigarette smoker; never regular cigarette smoker. The new harmonised binary variable took the value of one for current smoking and zero for all other categories.

Alcohol consumption

The Health Survey for England derived the frequency of alcohol drinking in the past twelve months and used the following categorisation structure: almost every day; five or six days a week; three or four days a week; once or twice a week; once or twice a month; once every couple of months; once or twice a year; not at all in the last 12 months/non-drinker. In the Thai Health-Risk Transition study dataset, the variable had the following categorisation structure: occasional social drinker; never; regular drinker; stopped. The new harmonised binary variable took the value of one for Health Survey for England categories “almost every day” and “five or six days a week” and for the Thai Cohort Study category “regular drinker”, and took the value of zero for all other categories.

Fruit and vegetables consumption

The Health Survey for England derived the grouped portions of fruit (including orange juice) and vegetables consumed yesterday and used the following categorisation structure: none; less than one portion; one portion or more but less than two; two portions or more but less than three; three portions or more but less than four; four portions or more but less than five; five portions or more but less than six; six portions or more but less than seven; seven portions or more but less than eight; eight portions or more. In the Thai Health-Risk Transition study dataset one continuous variable indicated the number of serves of vegetables per day and another continuous variable indicated the number of serves of fruit per day. The new harmonised binary variable took the value of one for Health Survey for England values that indicated less than three grouped portions of fruit and vegetables consumed yesterday and for Thai Cohort Study values that indicated less than three serves of vegetables or fruit per day, after the two original variables indicating vegetable and fruit consumption had been summed up.

Physical activity

The Health Survey for England derived a continuous variable indicating the average number of sessions of moderate to vigorous physical activity per week (including walking 30 minutes or more and sports / exercise sessions of 15 minutes or more, and excluding domestic activity). In the Thai Health-Risk Transition study dataset one continuous variable indicated the number of times of moderate exercise per week and another continuous variable indicated the number of times of strenuous exercise per week. The new harmonised binary variable took the value of one for Health Survey for England values that indicated less than two sessions of moderate to vigorous physical activity per week and for Thai Cohort Study values that indicated less than two times of moderate or strenuous exercise per week, after the two original variables indicating moderate and strenuous exercise per week had been added together.
